# Detection of EP300-ZNF384 fusion in patients with acute lymphoblastic leukemia using RNA fusion gene panel sequencing

**DOI:** 10.1007/s00277-020-04251-8

**Published:** 2020-09-26

**Authors:** Yu Jing, Yan-Fen Li, Hua Wan, Dai-Hong Liu

**Affiliations:** 1 Department of Hematology, Chinese People’s Liberation Army General Hospital, 100853 Beijing, China; 2 Beijing USCI Medical Laboratory, Beijing, 100195 China

**Keywords:** EP300, ZNF384, Fusion, Acute lymphoblastic leukemia, RNA sequencing

## Abstract

EP300-ZNF384 fusion is a rare recurrent cytogenetic abnormality associated with B cell acute lymphoblastic leukemia (B-ALL), which was rarely studied in Chinese patient cohort. Here, we used a customized RNA fusion gene panel to investigate gene fusions in 56 selected acute leukemia patients without conventional genetic abnormalities. Two EP300-ZNF384 fusion forms were detected in ten cases, which were in-frame fusions of *EP300* exon 6 fused with exon 3 or 2 of *ZNF384*. The fusions led to the lack of most functional domains of EP300. We firstly reported EP300-ZNF384 fusion in a mixed-phenotype acute leukemia (MPAL) patient whose CD33 and CD13 were negative. The rest nine B-ALL patients with EP300-ZNF384 fusion expressed CD33 and/or CD13. Fifty-six percent of B-ALL patients (5/9) with EP300-ZNF384 fusion were positive with CD10. After the diagnosis of EP300-ZNF384 fusion, 70% of the patients achieved remission after chemotherapy. Our observations indicated that EP300-ZNF384 fusion consists of a distinct subgroup of B-ALL with a characteristic immunophenotype. These patients are sensitive to current chemotherapy regimen and have an excellent outcome.

**Electronic supplementary material:**

The online version of this article (10.1007/s00277-020-04251-8) contains supplementary material, which is available to authorized users.

## Introduction

B cell acute lymphoblastic leukemia (B-ALL) is a clonal heterogeneous disease caused by the abnormal differentiation and maturation of B lymphoid progenitor cells, accompanied by abnormal proliferation. Its diverse biological characteristics result in clinical heterogeneity. Most patients with B-ALL can be detected with recurrent molecular genetic abnormalities, which could aid in clinical diagnosis and therapeutic decision. Some of the recurrent molecular genetic abnormalities, those associated with similar clinical phenotype, have been defined as subtypes in the WHO typing recommendations [1]. Increasing applications of next-generation sequencing (NGS) in the research on B-ALL have helped scientists and clinicians to grasp more new hidden pathogenic genetic events and then define new molecular subtypes. For example, ph-like B-ALL with tyrosine kinase-related gene fusion, and B-ALL with internal amplification of chromosome 21 (iMAP21) are newly defined high-risk subtypes with poor prognosis. However, owing to the high genetic heterogeneity of B-ALL, novel genetic subtypes and the clinical significance of some recurrent genetic abnormalities are largely underestimated.

The EP300-ZNF384 fusion gene is a newly identified recurrent form in B cell B-ALL, which has been reported with less than 50 B-ALL cases around the world. The EP300-ZNF384 fusion is formed by t(12;22)(p13;q13). The *EP300* gene is located at 22q13, encoding the E1A binding protein P300, a histone acetyltransferase (HAT), which can catalyze the acetylation of multiple substrates such as histone. It could interact with multiple transcription factors, and regulate cell growth, differentiation, cell cycle, and maintenance of genomic stability [2, 3]. The *ZNF384* gene is located at 12p13 and encodes a transcription factor C2H2 zinc finger protein. Other fusion genes involved *EP300*, *ZNF384* and dozens of other genes have been reported in acute myeloid leukemia (AML) and B-ALL [4–11]. Functional studies proved that ZNF384 fusion could block the differentiation of early B lymphocytes and induce acute leukemia in mouse models [12]. The B-ALL patients with EP300-ZNF384 were found to exhibit a special phenotype of weak CD10 and aberrant expression of CD13 and/or CD33 [6, 8, 13].

In this study, we used a customized RNA fusion gene panel test to screen for gene fusions in patients with acute leukemia. Ten patients were detected with EP300-ZNF384 fusion in 56 patients negative for popular gene fusions. The similar immunophenotype and response to chemotherapy indicated that the EP300-ZNF384 fusion could be defined as a novel subtype of B-ALL.

## Materials and methods

### Patient selection

Patients diagnosed with acute leukemia in our center were recruited from April 2016 to August 2018. Bone marrow specimens from 56 selected patients, who were negative for conventional gene fusions determined by multiple-PCR, were enrolled in this study (supplementary table 1). All the patients were diagnosed according to the WHO 2008 criteria for lymphoid tumors. Related clinical information was also collected. Informed consent of all patients was obtained according to the Declaration of Helsinki, and the study was approved by the Ethics Committee of Chinese People’s Liberation Army General Hospital.

### Targeted RNA sequencing

Total RNAs from the marrow samples were extracted by TRIzol reagent (Invitrogen, USA). Nanodrop 2000 spectrophotometer (Wilmington, USA) was used to measure the quantity and quality of the extracted RNA. Reverse transcription was performed according to the manufacture’s instruction by PrimeScript^TM^ RT reagent Kit (Takara, Japan). One hundred nanograms of sheared cDNAs was subjected to library construction with MGIEasy universal DNA library kit (MGI, China), then followed by hybrid capture using SureSelectQXT Reagent kit (Agilent, USA). Library quality and concentration were assessed by LabChip® GX Touch™ nucleic acid analyzer (PerkinElmer, USA) and Qubit fluorometer 3.0 (Life Technologies, USA), respectively. The qualified libraries were sequenced with 2 × 100 bp paired-end reads on a MGISEQ-2000 (MGI, China) platform.

### Bioinformatics analysis

The raw reads containing adaptor sequence and the reads with low quality (> 50% of bases whose Q scores were ≤ 10%) were filtered. The clean data were mapped to human reference genome hg19/GRCh37 using HISAT2 version 2.0.3-beta. Gene fusions were detected by fusionmap (10.0.1.29) [14] and STAR-Fusion Release v1.5.0 [15], followed by a customized filter strategy. (1) Briefly, the relative positions of the aligned paired sequences were compared to find the possible structural variation. The abnormally aligned sequences were segmented, and aligned by a more relaxed alignment method to map the sequences to possible positions to determine the final alignment position and direction. (2) To determine the positions of the breakpoint, the candidate final alignment positions were marked, and then the sequences were realigned. Only candidate breakpoints with high alignment quality and with supporting reads covering the points were retained. (3) Breakpoint correction was performed based on alignment scores, sequencing quality, and number of supported sequences. False positives were then filtered by tag reads covering the breakpoints, and paired reads supporting the break points.

### Fusion confirmation

Fusions detected in our cohort were confirmed using fluorescence in situ hybridization (FISH) and reverse transcription–PCR (RT-PCR) followed by Sanger sequencing. FISH analyses were performed using dual-color probes EP300 and ZNF384 (Guangzhou LBP Medicine Science and Technology, China) according to the manufacturer’s instruction. The analyses were performed using a Zeiss Axioplan 2 microscope (Carl Zeiss AG, Oberkochen, Germany) and the CytoVision software (Leica Biosystems, Nussloch, Germany). For each patient, 200 interphase cells were analyzed.

For detection of the EP300-ZNF384 fusion transcript, RT-PCR was performed using PrimeScript^TM^ RT reagent Kit (Takara, Japan) and TransTaq® HiFi DNA Polymerase (Transgen, China). Two forms of EP300-ZNF384 fusion were amplified with the same forward primer (5′-AATCAGATGCCGACACAACC-3′) and reverse primer (5′-CAGCAAGGTGGGGTAGTGAG-3′). The resulting 321 bp and 261 bp amplicons were confirmed by Sanger sequencing (Sangon Biotech, China).

### Cytogenetics and immunophenotypic analysis

Conventional G-banded chromosomal analysis was performed on unstimulated 24 h and 48 h bone marrow aspirate cultures. At least twenty metaphases were analyzed, and the results were reported using the 2009 International System for Human Cytogenetics Nomenclature (ISCN 2009).

Bone marrow aspirate specimens were subjected to standard ten-color flow cytometry immunophenotype analysis, and antibodies against the following antigens were used: CD3, CD7, CD10, CD13, CD14, CD15, CD11b, CD16, CD19, CD20, CD22, CD33, CD34, CD38, CD56, CD58, CD64, CD79a, CD117, CD123, HLA-DR, myeloperoxidase (MPO), and terminal deoxynucleotide transferase (TDT) (BD Biosciences, USA). Cytochemical stains for myeloperoxidase and non-specific esterase were performed on all diagnostic bone marrow aspirate smears.

## Results

### Detection of EP300-ZNF384 fusion in patients

**Fig. 1:**
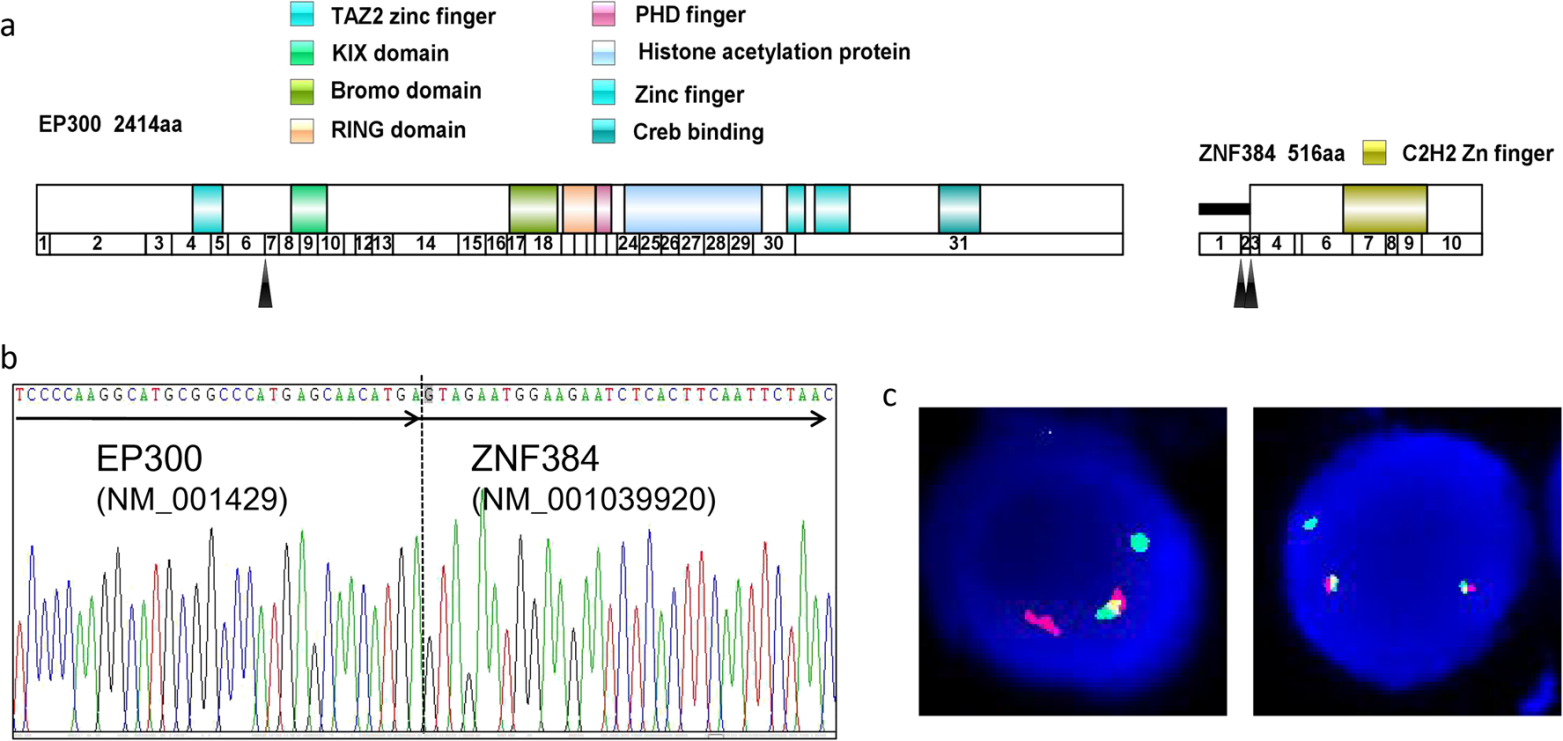
Structure and sequence of EP300-ZNF384 fusion. **a** Structural and functional domains of wild-type proteins of EP300 and ZNF384. Arrows indicated breakpoints of the wild-type proteins. **b** Sanger sequence of the fusion breakpoint between EP300 exon 6 and ZNF384 exon 3. **c**. EP300-ZNF384 fusion was confirmed by using dual-color FISH probes in interphase nuclei. The green signals were EP300 probes and the red signals were ZNF384 probes. The resulting yellow signal by tandem of red and green signals represented the EP300-ZNF384 fusion

The custom-designed panel, containing 225 genes, covered 1.1 Mb of the human genomes, which was designed for detection of known and unknown fusions at mRNA level. Among the 56 cases, 10 patients were identified with EP300-ZNF384 fusions. The putative fusions were validated by FISH and Sanger sequencing (Fig. 1 and Supplementary Table 2). There were two fusion isoforms in ten cases. The majority of patients (7/10, 70%) had a break point at EP300 exon 6 and ZNF384 exon 3, and one patient (1/10, 10%) at EP300 exon 6 and ZNF384 exon 2 (Fig. 1 and Tables 1 and 2). The remaining two had both isoforms. The resulting EP300 truncates eliminated the HAT and bromo domains, which was reported to reduce HAT activity and binding of acetylated proteins [13]. For ZNF384, the most part of the protein was retained in both isoforms.

**Table 1:** Clinical and genetic characteristics of acute leukemia with EP300-ZNF384 fusion

Case	Gender	Age (years)	Samples obtained at	Category	Initial WBC (*10^9^/L)	BM blast	Karyotype	Immunophenotype
1	Female	28	Rel (1st)	B-ALL	3.5	76%	46,XX[10]	CD19+/CD34+/CD22+/CD38+/CD58+/CD33+/CD123+/HLA-DR+/CD10-/CD20-/CD13-/CD15-
2	Female	63	Dx	B-ALL	12.83	89.2%	46,XX[5]	CD19+/CD34+/CD33+/HLA-DR+/CD79a+partial/CD38+/CD58+/CD22+/CD13+/CD10-/CD20-/MPO-/CD3-
3	Female	47	Dx	B-ALL	4.86	87.2%	46,XX[5]	CD19+/CD34+/CD38+/CD33+/CD22+/CD58+/HLA-DR+/CD79a+/CD20-/MPO-/CD15-/CD10-/CD13-/CD3-
4	Female	19	Dx	B-ALL	8.65	86%	45,X,-X[9] /46,XX[26] /Subdiploid[7]	CD19+/CD34+/CD38+/CD33+/CD22+/CD58+/HLA-DR+/CD79a+/CD15+/CD20-/MPO-/CD13-/CD10-/CD3-/CD117-
5	Female	43	Dx	B-ALL	2.7	79.2%	46,XX[20]	CD19+/CD34+/CD38+/CD22+/CD58+/CD10+partial/MPO+/HLA-DR+/CD79a+/CD20-/CD15-/CD33-/CD13-/CD16-/CD11b-/CD14-/CD64-/CD3-
6	Male	17	Dx	B-ALL	10.91	79.6%	46,XY,9qh+[20]	CD19+/CD34+/CD38+/CD33+/CD22+/CD13+/CD58+/CD10+/HLA-DR+/CD79a+/CD20-/MPO-/CD3-
7	Male	20	Dx	B-ALL	21.77	96%	46,XY[5]	CD19+/CD34+/CD38+/CD33+/CD22+/CD58+/CD10+/HLA-DR+/CD79a+/CD20-/MPO-/CD15-/CD13-/CD123-/IgM-/CD3-
8	Male	32	Rel (2^nd^)	B-ALL	2.67	97.2%	46,XY[20]	CD19+/CD34+/CD10+/CD22+/CD38+/CD58+/CD13+ partial/CD20-
9	Male	27	Dx	B-ALL	41.34	96%	46,XY[20]	CD19+/CD34+/CD38+/CD10+/CD22+/CD13+/CD58+/CD33+/HLA-DR+/CD79a+/CD20-/MPO-/CD3-
10	Male	38	Dx	B-ALL	118	93.6%	46,XY,t(11;12) (p11;p13)	CD19+/CD34+/CD38+/CD58+/CD22+/CD13+/CD33+/CD79a+/CD10+ partial/CD20-/MPO-/CD3-

*Dx*, diagnosis; *Rel*, relapse; *WBC*, white blood cell; *BM*, bone marrow

**Table 2:** Clinical and genetic characteristics of acute leukemia with EP300-ZNF384 fusion (continuation of Table 1)

Case	Fusion	Breakpoint	Treatment	Chemotherapy regimen	CR	Current status	Follow-up from determination of EP300-ZNF384 fusion (months)	Follow-up from diagnosis (months)
1	EP300-ZNF384	Ex6-Ex3	Chemotherapy, Allo-HSCT	VDCLP	Yes	Alive	26	84
2	EP300-ZNF384	Ex6-Ex3	Chemotherapy	VDCLP	Yes	Alive	30	30
3	EP300-ZNF384	Ex6-Ex3	Chemotherapy, Allo-HSCT	VDCLP	Yes	Alive	24	24
4	EP300-ZNF384	Ex6-Ex3, Ex6-Ex2	Chemotherapy	VDCLP-like	Yes	Alive	19	19
5	EP300-ZNF384	Ex6-Ex3	Chemotherapy	MAVP	No	Died	1	1
6	EP300-ZNF384	Ex6-Ex3, Ex6-Ex2	Chemotherapy	VDCLP	Yes	Alive	20	20
7	EP300-ZNF384	Ex6-Ex3	Chemotherapy, CarT, Allo-HSCT	VDCLP	No	Died	21	21
8	EP300-ZNF384	Ex6-Ex2	Chemotherapy, CarT, B-ALLo-HSCT	VACLP	No	Died	35	83
9	EP300-ZNF384	Ex6-Ex3	Chemotherapy, Allo-HSCT	VDCLP	Yes	Alive	36	36
10	EP300-ZNF384	Ex6-Ex3	Chemotherapy, Allo-HSCT	VDCLP	Yes	Alive	46	46

*CR*, complete remission; *Ex*, exon; *Allo-HSCT*, allogeneic hematopoietic stem cell transplantation; *CarT*, chimeric antigen receptor (CAR) T cell therapy; *VDCLP*, vincristine, daunorubicin, cyclophosphamide, L-asparaginase, and prednisone; *MAVP*, mitoxantrone, cytarabine, vincristine, and prednisone; *VACLP*, vincristine, cytarabine, cyclophosphamide, L-asparaginase, and prednisone

### Clinical characteristics of the patients with EP300-ZNF384 fusion

The clinical features of these 10 patients are summarized in Tables 1 and 2. The 10 patients included five females and five males, with a mean age of 33.4 (range from 17 to 63 years), including seven adolescent/young adults (AYA, 16–39 years) and three adults over 39 years. Nine patients were diagnosed as B-ALL, and one as mixed-phenotype acute leukemia (MPAL). The eight EP300-ZNF384 fusion patients were detected at the initial diagnosis, and the other two were at relapse. Their initial white blood cell (WBC) counts ranged from 2.67 × 10^9^ to 118 × 10^9^ with mean of 22.7 × 10^9^. The mean blast count (BM) was 88% (range from 76 to 97.2%).

The immunophenotypes were assessed in EP300-ZNF384 fusion cases. CD19 (10/10), CD22 (9/9), CD34 (10/10), CD38 (10/10), CD58 (10/10), and HLA-DR (8/8) were found to be positive in all tested cases. All cases were negative for CD3 (9/9) and CD20 (10/10). Half (5/10) of EP300-ZNF384 fusion-positive patients were positive for CD10 (CD10+), while the rest were found to have a weak expression or negative. All the males (5/9) of the B-ALL patients with EP300-ZNF384 fusion-positive were CD10+, and the females were CD10−. Five and eight patients with EP300-ZNF384 fusion genes exhibited expression of myeloid markers CD13 and CD33, respectively.

The conventional cytogenetic features of the EP300-ZNF384 fusion positive patients are also summarized in Tables 1 and 2. Seven out of ten patients showed a normal karyotype by conventional G-banding cytogenetic analysis. The predicted t(12;22)(p13;p13) chromosomal translocations for EP300-ZNF384 were not detected in these samples (Tables 1 and 2). This suggested that translocation leading to EP300–ZNF384 was under the detection limit of conventional G-banding, which was similar to earlier reports [7, 16].

### Treatments and outcomes

Eight of ten (80%) patients with EP300-ZNF384 fusion received VDCLP or VDCLP-like regimen induction chemotherapy. Seven patients (7/8, 87.5%) achieved complete remission (CR). The remaining two patients (one MPAL and one B-ALL) did not reach CR after MAVP or VACLP induction chemotherapy. Six of ten (60%) also underwent allogeneic hematopoietic stem cell transplantation (Allo-HSCT) including two that also received chimeric antigen receptor T cell therapy (CarT) (Tables 1 and 2).

At the time of last follow-up, 3 patients died, including one MPAL and two B-ALL patients with CarT. The mean survival was 19 months (range: 1–35 months) from the fusion determined (Tables 1 and 2). The MPAL case died 1 month after the diagnosis. The remaining alive cases were B-ALL patients. Taken together, our result showed that patients with MPAL had a much worse prognosis than those with B-ALL.

## Discussion

Gene fusion has proven to be an important clinical marker for the management of patients with acute leukemia, not only for diagnosis but also for predicting treatment outcomes, and selection of appropriate treatment methods. Conventional detection technologies include karyotype analysis, FISH, and RT-PCR. However, a large number of gene fusions are under the detection limits of karyotyping and the interpretations of FISH signals are often confusing and subjective. For instance, EP300 and ZNF384 genes were located near the telomeres of their respective chromosomes, so the EP300-ZNF384 fusion was technically difficult to be detected [7, 16]. RT-PCR is regarded as a sensitive test, but is only able to detect gene fusions corresponding to pre-designed primer sets and could not detect novel partner genes and rare breakpoints. Whole transcriptome sequencing has been used to investigate unknown cytogenetic alterations in B-ALL. However, the high sequencing cost hampered its clinical application. Targeted RNA fusion-related gene panel sequencing could be more efficient in clinical applications. Reliable gene fusions could be detected with higher performance price ratio [17, 18]. The RNA fusion gene panel used in this study, covered 225 genes, could detect over 900 fusion isoforms, and reached a high validation rate. Among the 56 patients without conventional gene fusion in this study, 10 were detected as EP300-ZNF384 positive. Gocho et al. [16] first found EP300-ZNF384 fusion in two samples out of 55 selected pediatric Philadelphia chromosome-negative precursor B acute lymphoblastic leukemia (BCP-ALL) patients without conventional genetic abnormalities. The frequencies of EP300-ZNF384 fusion are 1.3% (2/152) in children and 5.7% (7/122) in adolescent/young adult (AYA)/adult of BCR-ABL1-negative pre-B-ALL patients [19]. The frequencies of total ZNF384-fusion genes in BCP-ALL are 4–8% in children [6, 8] and 7% in adults [8], and 17% in adolescents and young adults (15–24 years old) [9].

This is the first report of EP300-ZNF384 fusion in MPLA patients. Inconsistent with the B-ALL patients, the patient was negative for CD13 and CD33 expression. There were 50% patients (5/10) with positive expression of CD10 in this study, inconsistent with previous reports that the EP300-ZNF384 fusion gene-positive patients are mostly associated with CD10 dull or low expression [16, 19]. Interestingly, all the B-ALL males with EP300-ZNF384 fusion were CD10+, while the corresponding females were CD10−. Therefore, positive expression of CD33 and/or CD13 is a certain immunotyping characteristic of B-ALL patients with EP300-ZNF384 fusion, while the lack of CD10 in B-ALL patients may not be a typical phenotype.

In summary, a growing number of EP300-ZNF384 fusion cases have been discovered in AYA and adults with B-ALL and MPAL. B-ALL patients with EP300-ZNF384 fusion had certain immunotyping characteristics, such as the expression of CD33 and/or CD13. The conventional chromosome karyotype cannot reflect the cryptic abnormality of EP300-ZNF384. Although the probes are difficult to hybridize near teleomeres, the fusion has been confirmed by FISH in this study. Array CGH will be performed in our future study to verify copy number alteration of EP300 or ZNF384. Most patients with EP300-ZNF384 fusion could achieve CR after chemotherapy regimen, reflecting an ideal clinical outcome. All these further suggested that the B-ALL with EP300-ZNF384 fusion could be defined as a novel subtype with unique clinical and laboratory characteristics.

## Electronic supplementary material

ESM 1 (PDF 178 kb)

## Data Availability

The datasets used during the current study are available from the corresponding author on reasonable request.
