# Supplementary material for: Detection of EP300-ZNF384 fusion in patients with acute lymphoblastic leukemia using RNA fusion gene panel sequencing
Source: Ann Hematol. 2020 Sep 26;99(11):2611–7. doi: 10.1007/s00277-020-04251-8 (PMC7536166; doi:10.1007/s00277-020-04251-8)
Supplement: Supplementary file 1 — (PDF 178 kb) [file 277_2020_4251_MOESM1_ESM.pdf]

Legends to supplementary Tables

Supplementary-Table 1

Conventional gene fusion types detected by multiple-PCR. All the 56 case in this study was negative for the conventional gene fusions.

Supplementary-Table 2

Detection of EP300-ZNF384 fusion by FISH. Eight out of ten cases were confirmed with FISH probes, except case 5 and 7, of which there were no more bone marrow specimens left. The green signals were EP300 probes and the red signals were ZNF384 probes. The yellow signals and the red and green signals in close tandem represent overlapping red and green signals are EP300-ZNF384 fusion.

Supplementary-Table 1

**Conventional gene fusion types detected by multiple-PCR**
AML1/ETO
AML1/EVI1
AML1/MDS1
AML1/MTG16
AML1/PRDM16
BCR/ABL
BCR/PDGFRA
CBFB/MYH11

C-MAF/IGH
DEK/CAN
E2A/PBX1
FIPIL1/PDGFRα
HOX11/TCR
HOX11L2/TCR
MLL/AF10
MLL/AF17
MLL/AF4
MLL/AF6
MLL/AF9
MLL/ELL
MLL/ENL
MLL/MLL
MLL/PTD
MYO/FGFR1
NPM1/ALK
NPM1/RARA
NUP98/HOXA9
PLZF/RARA
PML/RARA

SET/CAN
SIL/TAL1
TEL/ABL
TEL/AML1
TEL/JAK2
TEL/PDGFRα
TEL/PDGFRβ
TLS/ERG

Supplementary-Table 2

Case	No. of cell with yellow color	Percent in 200 interphase cells	Figure
**1**	131	66%	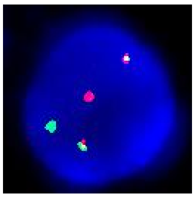
**2**	129	65%	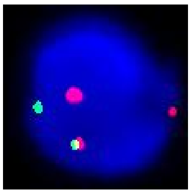
**3**	20	10%	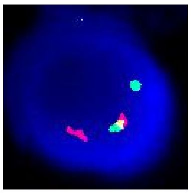

**4**	171	86%	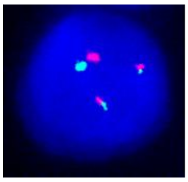
**6**	135	68%	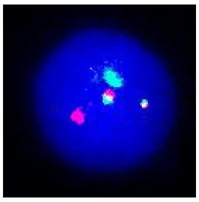
**8**	154	77%	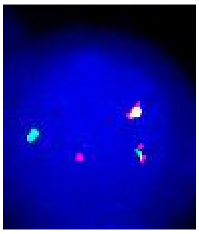
**9**	143	72%	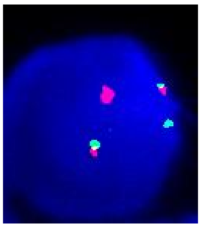
**10**	154	77%	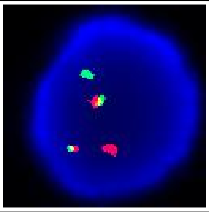
